# Model-based variables for the kinematic assessment of upper-extremity impairments in post-stroke patients

**DOI:** 10.1186/s12984-016-0187-9

**Published:** 2016-09-08

**Authors:** Alessandro Panarese, Elvira Pirondini, Peppino Tropea, Benedetta Cesqui, Federico Posteraro, Silvestro Micera

**Affiliations:** 1 The BioRobotics Institute, Scuola Superiore Sant’Anna, Viale R. Piaggio 34, 56025 Pontedera, Pisa Italy; 2 Bertarelli Foundation Chair in Translational Neuroengineering, Center for Neuroprosthetics and Institute of Bioengineering, School of Engineering, École Polytechnique Fédérale de Lausanne (EPFL), Lausanne, Switzerland; 3 Centre of Space Bio-medicine, University of Rome Tor Vergata, Rome, Italy; 4 Laboratory of Neuromotor Physiology, IRCCS Santa Lucia Foundation, Rome, Italy; 5 Rehabilitation Department Versilia Hospital, AUSL 12, Viareggio, Italy; 6 Bioengineering Rehabilitation Laboratory, Auxilium Vitae Rehabilitation Centre, Volterra, Italy

**Keywords:** Stroke, Robotic rehabilitation, Kinematics, Modeling

## Abstract

**Background:**

Common scales for clinical evaluation of post-stroke upper-limb motor recovery are often complemented with kinematic parameters extracted from movement trajectories. However, there is no a general consensus on which parameters to use. Moreover, the selected variables may be redundant and highly correlated or, conversely, may incompletely sample the kinematic information from the trajectories. Here we sought to identify a set of clinically useful variables for an exhaustive but yet economical kinematic characterization of upper limb movements performed by post-stroke hemiparetic subjects.

**Methods:**

For this purpose, we pursued a top-down model-driven approach, seeking which kinematic parameters were pivotal for a computational model to generate trajectories of point-to-point planar movements similar to those made by post-stroke subjects at different levels of impairment.

**Results:**

The set of kinematic variables used in the model allowed for the generation of trajectories significantly similar to those of either sub-acute or chronic post-stroke patients at different time points during the therapy. Simulated trajectories also correctly reproduced many kinematic features of real movements, as assessed by an extensive set of kinematic metrics computed on both real and simulated curves. When inspected for redundancy, we found that variations in the variables used in the model were explained by three different underlying and unobserved factors related to movement efficiency, speed, and accuracy, possibly revealing different working mechanisms of recovery.

**Conclusion:**

This study identified a set of measures capable of extensively characterizing the kinematics of upper limb movements performed by post-stroke subjects and of tracking changes of different motor improvement aspects throughout the rehabilitation process.

**Electronic supplementary material:**

The online version of this article (doi:10.1186/s12984-016-0187-9) contains supplementary material, which is available to authorized users.

## Background

Upper limb functions are altered in about 80 % of acute stroke survivors and in about 50 % of chronic post-stroke patients [1]. With the increasing of life expectancy, it has been estimated that stroke related impairments will be ranked to the fourth most important causes of adult disability in 2030 [2], prompting the need to design more effective diagnostic and rehabilitative tools [3, 4].

Together with more traditional and widely accepted clinical scales in the last two decades investigators have characterized post-stroke motor recovery also in terms of kinematic parameters extracted from hand and arm task-oriented movements [3, 5], which offer more objective measures of motor performance [6]. Indeed, clinical scales, whose reliability has often been questioned [7–9], may not be sensitive to small and more specific changes [10] and could be of limited use to distinguish different aspects of motor improvement [11, 12].

Previous robot-assisted clinical and pilot studies have proposed a large set of kinematic parameters to characterize motor improvements [5, 6, 11]. A few of them focused on finding a significant relationship between robotic measures collected longitudinally in post-stroke patients and clinical outcome measures, to increase acceptance of kinematic evaluation scales in practice [5, 6]. Too little effort, however, has been made to identify the different aspects of movement improvement, how they can be described by kinematic robot-based measures [11], and whether they may dissociate with respect to recovery time course and to training response [11].

Indeed the range of potential changes in limb trajectory during recovery is not known *a priori* [12] and might not be fully represented by a set of arbitrarily selected parameters extracted from limb trajectories, even if the parameters were chosen according to a certain number of study hypotheses or to significant relationships with clinical scales. Moreover, these variables can be highly correlated and, thus, redundant. Although redundancy can be tackled by data reduction algorithms, such as Principal Component Analysis (PCA) or Independent Component Analysis (ICA) [5, 6], incomplete representation of information might still remain an overlooked issue.

In the present study we aimed at devising a novel method for identifying a set of kinematic measures potentially capable of fully highlighting and tracking changes of different aspects of movement performance throughout the rehabilitation training. Instead of starting from a certain number of *a priori* hypotheses, we sought to find which variables were essential for modeling trajectories of post-stroke patients and were, thus, informative of kinematic features of upper limb movements. We then tested whether the identified kinematic parameters *i)* were capable of highlighting changes in movement performance, *ii)* were to some extent redundant, and *iii)* were informative of different factors of post-stroke motor impairment, such as paresis, loss of fractionated movement and somatosensation, and abnormal muscle tone [13].

## Methods

### Participants

The data from 12 patients (6 men 6 women) were included in this study. All patients experienced a single unilateral cerebrovascular accident. 6 patients (sub-acute, age 71.8 ± 5.4 years) were enrolled less than 40 days after stroke, and 6 patients (chronic, age 64.0 ± 12.9 years) between 5 and 142 months post-stroke. Participants’ Fugl-Meyer Assessment (FMA) scores [14, 15] were 20.5 ± 9.0 and 27.5 ± 8.7 for sub-acute and chronic patients, respectively. Table 1 reports a summary of the features related to all patients at the beginning of the therapy. Data related to the sub-acute patients and details on the rehabilitation protocol were already reported in a previous study [16].

**Table 1:** Summary of stroke patients inserted in the study

Sub-Acute Patients	Gender	Age yrs	Days elapsed from the accident	Dominance	Paretic Side	Stroke type	Location
Sub01	M	82	37	R	L	I	Right cortical-subcortical frontal
Sub02	F	66	29	R	L	I	Right Frontal-temporal-parietal
Sub03	M	70	27	R	L	I	Right cortical-subcortical precentral
Sub04	M	70	24	R	R	H	Left internal capsule
Sub05	M	72	14	R	L	I	Right cortical-subcortical parietal
Sub06	F	71	19	R	L	I	Right paramedian pontis
Chronic patients	Gender	Age yrs	Months elapsed from the accident	Dominance	Paretic Side	Stroke type	Location
Sub07	F	58	143	R	L	I	Right talamus
Sub08	F	67	139	R	L	H	Right talamus
Sub09	M	47	5	R	R	H	Left nucleus lenticolar
Sub10	F	61	5	R	L	H	Right thalamus capsular
Sub11	F	86	58	R	R	I	Left nucleus caudatus and thalamus
Sub12	M	65	8	R	L	H	Right fronto-parietal

Labels in the 2^nd^, 5^th^, 6^th^, and 7^th^ columns refer to: *F* Female, *M* Male, *L* Left, *R* Right, *H* Hemorrhagic, *I* Ischemic

Due to the limited sample size the inhomogeneity in the patient groups and in the treatment schedules (see Rehabilitation protocol), motor performances will not be compared between the two stroke survivor populations.

Seven neurologically intact age-matched subjects (5 men 2 women; age 72 ± 5 years) were also included in the study as control group (part of their data were published earlier [17]). Healthy participants exhibited normal ranges of motion and muscle strength and they did not show any functional disability.

### Rehabilitation protocol

**Fig. 1:**
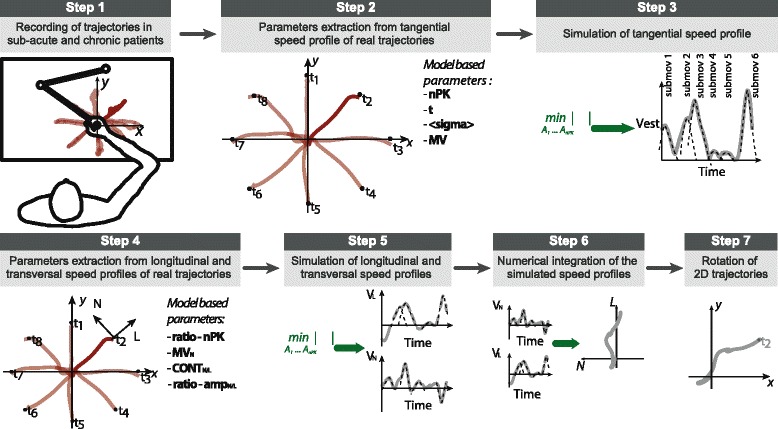
Schematic overview of the computational model for post-stroke trajectories simulation. (1) Endpoint kinematics of one pathological subject making point to point forward and backward movements from the center of the workspace to one of eight different targets equally spaced around a circle of 14 cm of radius assisted by InMotion2. (2) Kinematic parameters are extracted from the real trajectories of the post-stroke subjects. The tangential speed profile of real trajectories (for each movement direction and subject, separately for each group of patients and time of therapy) is analyzed to extract probability distributions of **nPK**, **<σ>**, **T**, and **MV**. (3) Based on the inferred probability distributions, tangential speed profiles of simulated trajectories are generated by solving a constrained optimization problem (Eq. 2). (4) The transversal and longitudinal speed profiles of real trajectories are analyzed to extract probability distributions of **ratio-amp**_**L**_, **ratio-amp**_**N**_, **ratio-nPK**, **MV**_**N**_, **CONT**_**L**_, and **CONT**_**N**_. (5) From the simulated tangential velocities and the inferred distributions of kinematic parameters, transversal and longitudinal speed profiles of simulated trajectories are generated, by solving an unconstrained optimization problem (Eq. 4). (6) The trajectories in the Cartesian space defined by the (L, N) axes (see step 4) are obtained from the speed profiles by numerical integration. (7) The generated trajectories are then rotated by means of a geometrical transformation to reproduce the point-to-point movements performed by post-stroke subjects during a *turn* in the InMotion2 coordinate frame system

The patients and the healthy subjects were instructed to make point-to-point reaching movements forward and backward from the center of the workspace to one of eight different targets equally spaced around a circle of 14 cm of radius (Fig. 1 step 1) assisted by InMotion2 (Interactive Motion Technologies, Inc. Cambridge, Massachusetts) [18]. When the subjects sequentially carried out all the 16 movements, they completed one full *turn*.

The rehabilitation protocol consisted of 45 min of robot-assisted therapy (at least 65 *turns* per sessions) five days per week. Sub-acute patients practiced for 6 weeks whereas chronic patients for 4 weeks. The difference in the treatment schedules was due to the organizational structure of the hospital in which the treatment was carried out. The robot provided assisting force when patients were not able to reach the targets, except for 2/3 *turns* per session (i.e., *assessing turns*) that were used to assess subjects’ motor performance. In this study, only the data related to these *assessing turns* and the data recorded without application of external force fields for the healthy subjects were included. The protocol was approved by the Local Ethical Committee (Comitato Etico Azienda Ospedaliera Universitaria di Pisa. Reference N°: 2754, year 2009).

Participants’ upper limb motor functions were evaluated before (*T*_*0*_) and after (*T*_*1*_) the rehabilitative treatment by an experienced physiatrist using clinical scales: FMA [14, 15], Modified Ashworth Scale (MAS) for shoulder and elbow [19], and Motricity index (MI) [20].

### Computational model

We devised a computational model able to simulate end-point planar trajectories of stroke patients for the given upper-limb rehabilitation protocol (see Rehabilitation protocol) starting from the knowledge of a limited number of kinematic parameters (*i.e., Model-based parameters*), whose values were specified according to the experimental data (*i.e.*, extracted from the real trajectories of the post-stroke patients included in this study). Indeed, simulated trajectories geometrically similar to those of stroke patients, and with comparable kinematic features, would imply that the *Model-based parameters* are a thorough and yet parsimonious way to represent the kinematic information contained in the trajectories of post-stroke subjects.

Unlike previous studies [21, 22], point-to-point movements were modeled specifying the complete trajectory on the plane. Indeed, unidimensional models are suitable for modeling healthy adult movements, which are approximately straight in the Cartesian space [21, 23], but inevitably they neglect to characterize the deviations from the *theoretical path* (*i.e.,* the straight line connecting the start and the target points), which are indeed important markers of motor improvements of post-stroke patients.

We started by modeling the time profile of a trajectory’s tangential speed *v*_*T*_*(t)*, as a sum of submovement curves (*i.e.*, single discrete movements that contribute to the resulting movement path, Fig. 1, step 3):

1 $$ {v}_T(t)={\displaystyle {\sum}_{i=1}^{\mathbf{nPK}}{A}_i{e}^{-{\left(\frac{t-{t}_i}{\sqrt{2}{\sigma}_i}\right)}^2}} $$

such that:

0 ≤ *t* ≤ ***t***,

$$ \frac{1}{\boldsymbol{t}}{\displaystyle {\int}_0^{\boldsymbol{t}}v(t)dt=MV,} $$

*E*[*σ*] = *<***σ>**.

These curves were assumed to be bell-shaped Gaussian profiles centered at time *t*_*i*_ with amplitude ***A***_***i***__*i*_ and duration proportional to *σ*_*i*_. Indeed, Krebs et al. [24] demonstrated that submovement speed profiles are remarkably similar across patients even though neurological damages are not, and can be appropriately approximated by nearly symmetrical β-functions or by Gaussian curves. The peak times, *t*_*i*_, in the tangential speed profile were randomly drawn from a uniform distribution *U* (0,**t**), with the constraint of being spaced at least by 2**<σ>** to avoid peak overlapping, and *σ*_*i*_ were randomly chosen such that *E*[*σ*] = **<σ>**.

This *v*_*T*_*(t)* profile model requires the specification of the number of submovements of which the entire movement is composed of i.e., *n* = −**nPK**, their average peak duration **<σ>**, the movement duration, **t**, and the average tangential speed, **MV**. To generate simulated *v*_*T*_*(t)* profiles specific for each patient group (sub-acute, chronic) and time point during the therapy (*T*_*0*_, *T*_*1*_), we first extracted the values of **nPK**, **<σ>**, **t**, **MV** from the trajectories recorded during the experiments (Fig. 1, step 2). In particular, **nPK** was obtained with the constraint that peaks amplitude had to be larger than the 10 % of the maximum speed amplitude [25] and **<σ>** was computed as the average σ of the Gaussian curves fitted to each tangential submovement.

We then estimated different (for each patient group and time point) probability distributions for these parameters (pooling data across repetitions subjects, and movement directions). A new random sample from each distribution was drawn each time a new simulated tangential speed profile had to be generated. The new simulated tangential speed profile was computed by solving a numerical optimization problem (constrained problem with the active set algorithm implemented in the function *fmincon*) in Matlab (Mathworks, Inc., MA, USA):

2 $$ \underset{A_1\dots {A}_{\mathbf{nPK}}}{ \min}\left|MV-\frac{1}{\mathbf{t}}{\displaystyle {\int}_0^{\boldsymbol{t}}v}\left(t,{A}_1\dots {A}_{\mathbf{nPK}}\right)dt\right| $$

subject to the constraint:

$$ {A}_i>0,\kern3.75em i=1,\dots, \mathbf{n}\mathbf{P}\mathbf{K} $$

The problem of generating simulated two-dimensional trajectories required the specification of end-point motion in two axes on the plane of movement. We chose the axis defined by the *theoretical path* and the corresponding orthogonal axis (the L and N axes respectively, in Fig. 1, step 4). The end-point motion was specified in terms of the projections of the velocity vector, ***v****(t)*, along these two axes: the longitudinal, *v*_*L*_*(t)*, and the transversal, *v*_*N*_*(t)*, speed profiles. This problem cannot be solved analytically because *v*_*T*_*(t)* did not contain information regarding the direction of the movement, thus a number of assumptions were made to obtain approximated solutions for *v*_*L*_*(t)* and *v*_*N*_*(t)*.

First similar to *v*_*T*_*(t),* we assumed that *v*_*L*_*(t)* and *v*_*N*_*(t)* were composed of a sum of Gaussian bell-shaped curves. Indeed, these curves represent submovements projected onto orthogonal directions: the longitudinal, L, and the transversal, N, axes (see Fig. 1, step 4). Moreover, the correlation between a fitted sum of Gaussian bell-shaped curves with the experimental longitudinal and transversal speed profiles was high (Pearson’s correlation: *ρ* = 0.75 ± 0.18).

Second we considered that a number of peaks in *v*_*T*_*(t)* could be the result of movements traveling predominantly in the longitudinal or transversal direction. To take this into account, two additional parameters were added to the model: **CONT**_**L**_ and **CONT**_**N**_, i.e. the number of peaks in *v*_*L*_*(t)* and *v*_*N*_*(t)*, respectively, producing a corresponding peak in *v*_*T*_*(t)*. **CONT**_**x**_ ($\mathrm{with}x\in \left\{L,N\right\}$) was calculated on the longitudinal or the transversal speed profile of each experimental trajectory as the sum of “contributing peaks”, i.e. peaks falling within a time interval as large as **σ** and centered on peaks of the tangential velocity. For *v*_*L*_*(t)* and *v*_*N*_*(t),* the number of peaks (i.e., **nPK**_**L**_ and **nPK**_**N,**_ respectively) were calculated following the same technique used for *v*_*T*_*(t)* considering separately the positive and the negative part of the speed profile, resulting in the estimation of positive and negative peaks, respectively.

Third **nPK**_**L**_ and **nPK**_**N**_ were assumed to be comparable because they had, on average, similar values in our dataset (except for sub-acute patients at *T*_*0*_*; p* = 0.04 Wilcoxon rank sum test, significance level α = 0.05) and were computed from the **ratio-nPK** (ratio between the number of peaks in the longitudinal and in the tangential velocity profiles). However, should this assumption be falsified in a new cohort of patients larger than ours, the model could be easily modified to include both **nPK**_**L**_ and **nPK**_**N**_ in the *Model-based parameters*.

The longitudinal and transversal speed profiles can be expressed similarly to *v*_*T*_*(t)* (see Eq. 1) with *t*_*i*_^(*x*)^ and ***A***_***i***__*i*_^(*x*)^ the central instants and amplitudes of the peaks in the speed profile with *x* ∈ {*L*, *N*}. A number of **CONT**_**x**_ central instants were the same at which peaks in *v*_*T*_*(t)* occur, whereas the remaining **nPK**_**x**_ - **CONT**_**x**_ were randomly chosen in [0, **t**], with a constraint on peak-to-peak distance similar to that for *v*_*T*_*(t)*:

3 $$ {v}_x(t)={{\displaystyle {\sum}_{i=1}^{\mathbf{nP}{\mathbf{K}}_{\mathbf{x}}}{A}_i^{(x)}e}}^{-{\left(\frac{t-{t}_i^{(x)}}{\sqrt{2}{\sigma}_i}\right)}^2} $$

such that:

0 ≤ *t* ≤ ***t***,

$$ \frac{1}{\boldsymbol{t}}{\displaystyle \underset{0}{\overset{\boldsymbol{t}}{\int }}}v(t)dt=M{V}_x, $$

*E*[*σ*] = ***<σ>***.

with *x* ∈ {*L*, *N*}. The computation of the two velocities can be reduced to a single unconstrained optimization problem to find the amplitudes $\left[{A}_i^{(L)}\dots {A}_{{\mathbf{nPK}}_{\mathbf{L}}}^{(L)},\kern0.5em {A}_i^{(N)}\dots {A}_{{\mathbf{nPK}}_{\mathbf{N}}}^{(N)}\right]$ (see Fig. 1 step 5). Initial values for these amplitudes were set by using two additional parameters: **ratio-amp**_**L**_, the ratio between average longitudinal and average tangential peak amplitude, and **ratio-amp**_**N**_ (i.e., same parameter with the average transversal peak amplitude in the numerator). Specifically, the function to minimize was:

4 $$ \underset{\left[{A}_1^{(l)}\dots {A}_{{\mathbf{nPK}}_{\mathbf{L}}}^{(l)},{A}_1^{(n)}\dots {A}_{{\mathbf{nPK}}_{\mathbf{N}}}^{(n)}\right]}{ \min}\alpha \left|M{V}_l-\frac{1}{\mathbf{t}}{\displaystyle {\int}_0^{\boldsymbol{t}}{v}_l}\left(t,{A}_1^{(l)}\dots {A}_{{\mathbf{nPK}}_{\mathbf{L}}}^{(l)}\right)dt\right|+\beta \left|M{V}_n-\frac{1}{\mathbf{t}}{\displaystyle {\int}_0^{\boldsymbol{t}}{v}_n}\left(t,{A}_1^{(n)}\dots {A}_{{\mathbf{nPK}}_{\mathbf{N}}}^{(n)}\right)dt\right| $$

with *α* and *β* two parameters (*α, β* ≤ 1) whose values were chosen to obtain a solution *v* = (*v*_*L*_*(t)*, *v*_*N*_*(t)*) meeting the requirements for MV_L_ and MV_N_ (i.e., average *v*_*L*_*(t)* and *v*_*N*_*(t)*, respectively) at different compliance levels. In our simulations we used *α =* 0.8 and *β* = 0.2.

The generation of *v*_*L*_*(t)* and *v*_*N*_*(t)* starting from the peaks (timing and amplitude) in the tangential velocity (that indicate changes in 2D movement direction and then determine 2D shape) allows the model to preserve the link between longitudinal and transversal speed components which was present also in the real trajectories (see Additional file 1: Figure S1 for a comparison of correlation values between longitudinal and transversal components in simulated and real trajectories).

As for the tangential speed the values of the six parameters required for the generation of simulated *v*_*L*_*(t)* and *v*_*N*_*(t)* (**MV**_**N,**_**CONT**_**L**_, **CONT**_**N**_, **ratio-amp**_**L**_, **ratio-amp**_**N,**_**ra**t**io-nPK**) were extracted from the trajectories recorded during the experiments, and subsequently pooled to estimate probability distributions, which were specific for each patient group and time point during the therapy (Fig. 1, step 2 and step 4). **MV**_**L**_ was not directly estimated from the experimental trajectories but calculated as the ratio between the distance center-target, **D** = 14 cm, and the overall (experimental) duration of the movement, **t**.

Once the speed profiles were computed (by solving an unconstrained optimization problem with the active set algorithm implemented in the function *fminsearch* of Matlab) they were numerically integrated over time to obtain the trajectory components along the longitudinal and transversal directions: *x*_*L*_*(t)* and *x*_*N*_*(t)*, respectively (see Fig. 1, step 6). Finally, the two trajectory components were rotated to express the simulated end-point trajectory in the InMotion2 coordinate frame (see Fig. 1, step 7).

Sensitivity analysis was performed to demonstrate the stability of the model: for both group of patients and time points (*T*_*0*_ and *T*_*1*_) a parameter at a time was varied (ten variations equally distributed across its probability distribution) while keeping the other parameters fixed at the average value of their probability distribution. Results of sensitivity analysis were comparable to those found with the parameters values chosen showing that the model was robust for variation of the parameters (see Additional file 2: Figure S2).

### Model validation

The mean Euclidean distance normalized by the total distance traveled during the reaching movement was used to assess the geometrical similarity between real and simulated trajectories ***E***_*RS*_. Simulated trajectories were considered similar to the real ones if the range of variation of ***E***_*RS*_ was comparable to ***E***_*I*_^*(R)*^, i.e., the intrinsic trajectory variability (inter-subject and intra-subject) of real curves.

We then validated the ability of the simulated trajectories to capture significant kinematic features of real movements by comparing the values of an extensive set of additional kinematic parameters not used by the model, computed on both simulated and real trajectories. This set of parameters was chosen to extensively characterize longitudinal and transversal speed profiles and to check the suitability of the optimization strategies used to simulate the speed profiles (i.e., tangential, longitudinal, and transversal) and, consequently, the trajectories. The following parameters (*i.e., Evaluation parameters*) were considered: **MV**_**L**_, average longitudinal speed; **A** average amplitude of peaks in *v*_*T*_*(t)*; **A**_**L**_**–pos** and **A**_**L**_**–neg** (**A**_**N**_**–pos** and **A**_**N**_**–neg**) average amplitude of positive and negative peaks for *v*_*L*_*(t)* (*v*_*N*_*(t)*); **nPK**_**L**_**–pos** and **nPK**_**L**_**–neg** (**nPK**_**N**_**–pos** and **nPK**_**N**_**–neg**) the number of positive and negative peaks for *v*_*L*_*(t)* (*v*_*N*_*(t)*); **CONT**_**N**_**-amp** the ratio between the average transversal and tangential peak amplitudes, computed only for “contributing” peaks; **CONT**_**L**_**-amp** similar parameter for *v*_*L*_*(t)*; **MD** the mean absolute value of the distance between the actual trajectory and the *theoretical path*; **overlap** the overlapping area between two submovements (intersection between the two Gaussian curves describing the submovements normalized by the sum of the two areas).

As a further validation of the model we tested the similarity between shoulder and elbow angular excursions computed from the simulated and the real trajectories using the two-link model for upper-limb movements introduced in [25]. Indeed, previous studies evaluated shoulder and elbow angular excursions during different tasks and reported an abnormal joint coupling in post-stroke patients that reduces during rehabilitative treatment [26–28].

Joint angles were estimated as:

$$ \begin{array}{c}\hfill {\uptheta}_1={ \tan}^{-1}\left(\mathrm{y},\mathrm{x}\right)-{ \tan}^{-1}\left(\mathrm{k},{\mathrm{x}}^2+{\mathrm{y}}^2+{\mathrm{l}}_1^2-{\mathrm{l}}_2^2\right)\hfill \\ {}\hfill {\uptheta}_2={ \tan}^{-1}\left(\mathrm{k},{\mathrm{x}}^2+{\mathrm{y}}^2-{\mathrm{l}}_1^2-{\mathrm{l}}_2^2\right) + {\uptheta}_1\hfill \end{array} $$

where θ_1_ and θ_2_ are respectively shoulder and elbow joint angles $\mathrm{k}=\sqrt{{\left({\mathrm{x}}^2+{\mathrm{y}}^2+{\mathrm{l}}_1^2+{\mathrm{l}}_2^2\right)}^2-2\Big[{\left({\mathrm{x}}^2+{\mathrm{y}}^2\right)}^2+{\mathrm{l}}_1^4+{\mathrm{l}}_2^4}\Big],-\uppi \le { \tan}^{-1}\le \uppi, {\mathrm{l}}_1$ is the upper arm length, and l_2_ the lower arm length. Values of the parameters l_1_ and l_2_ were estimated both for simulated and real trajectories from the measurements of the 50th percentile for U.S. males [29]: l_1_was estimated as 0.282 m; whereas l_2_ was calculated as the sum of forearm length (0.254 m) the distance from the wrist to the handle (0.076 m), and the handle robot radius (0.03 m).

Joint angular excursions computed from the simulated and the real trajectories were compared using ***d***_*%*_ (the average distance between two angular trajectories resampled to the same number of time points and normalized by the maximum angular excursion of the real trajectory).

### Investigation of underlying factors of motor recovery

Finally we inspected the *Model-based parameters* both to evaluate redundancy among the parameters and to find whether longitudinal variations in these variables might have been explained by a restricted number of underlying (and unobserved) factors, putatively reflecting different aspects of motor recovery [11]. For this purpose, we performed a Factor Analysis (FA) with Maximum Likelihood extraction and promax rotation method on the *Model-based parameters*. These were pooled from all movement directions and repetitions for healthy subjects and patients at each day of rehabilitation. The number of retained factors was selected on the basis of “cleanliness of factor structure”, and both the individual (>5 %) and the cumulative percentage (≥70 %) of total variance explained [30]. For each factor, the parameters with loadings >0.6 were clustered together [30]. Variables with loadings not exceeding the threshold for none of the factors were named “shared”. To determine the time course of each factor along the rehabilitation process, for each patients group separately, we fitted the data to different functions: straight line, exponential decay, and double-exponential decay. The best fit was then selected among these three functions according to the Bayesian Information Criterion (BIC) [31].

### Statistical analyses

Comparisons between *T*_*0*_ and *T*_*1*_ clinical scores and kinematic parameters were made with a Wilcoxon signed-rank test (significance level α = 0.05), for the two groups of patients separately. Parameters from real and simulated trajectories were analyzed separately. ***E***_*RS*_ values were compared with the intrinsic variability of the real trajectories, ***E***_*I*_^*(R)*^, for both groups of patients and time points (*T*_*0*_ and *T*_*1*_) using a Wilcoxon rank sum test (significance level, α = 0.05).

## Results

### Simulated trajectories reliably approximated real trajectories of post-stroke subjects

**Fig. 2:**
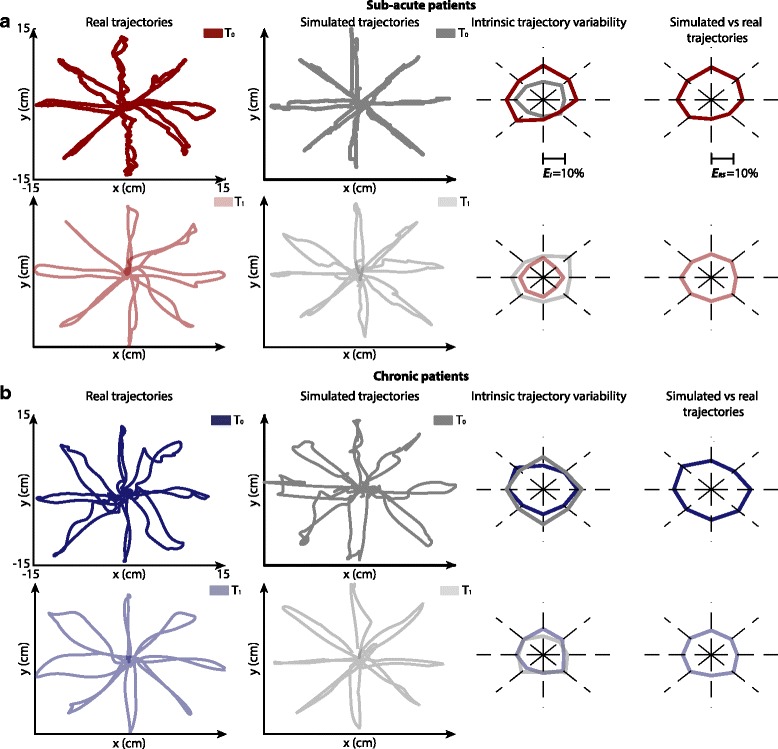
Real and simulated trajectories: Cartesian space. **a** Results for sub-acute patients. Real and simulated trajectories (*first and second columns respectively*) at *T*_*0*_ (*first row*) and *T*_*1*_ (*second row*) for a representative subject and for one repetition of the simulation. On the third columns the angular plots of the average normalized Euclidean distance among repetitions of the model (*grey line*, ***E***_*I*_^*(S)*^) and among subjects (*red line*, ***E***_*I*_^*(R)*^) for the 8 directions of movements. On the fourth columns the angular plots of the average normalized Euclidean distance between repetitions of the model and subjects for the 8 directions of movements (***E***_*RS*_). **b** Results for a representative chronic patient, same organization of sub-acute patient

Simulated trajectories were generated for sub-acute and chronic patients both at time *T*_*0*_ and *T*_*1*_ (Fig. 2). As explained in *Methods* (Computational model) our aim was not to precisely reconstruct real trajectories, but to generate simulated trajectories consistent with the real ones. Indeed, simulated trajectories showed significant geometrical similarity with the real trajectories. In fact, the range of variation of ***E***_*RS*_ was comparable to the intrinsic trajectory variability of real curves, ***E***_*I*_^*(R)*^, both at *T*_*0*_ and *T*_*1*_. The average ***E***_*RS*_ (across movement directions and subjects) was 14.52 ± 0.78 % at *T*_*0*_ and 13.02 ± 0.52 % at *T*_*1*_, for sub-acute patients, and 16.88 ± 1.43 % at *T*_*0*_ and 13.05 ± 1.25 % at *T*_*1*_, for chronic patients. Comparable ranges were confirmed by statistical tests. Indeed, only ***E***_*RS*_ for sub-acute patients at *T*_*1*_ was higher than ***E***_*I*_^*(R)*^ (*p* = 0.008).

Interestingly ***E***_*I*_^*(R)*^ decreased after therapy, showing a reduction of performance variability within each group of patients, which was correctly captured by our model in the case of chronic patients (***E***_*I*_^*(R)*^: 15.71 ± 2.87 % at *T*_*0*_ and 9.90 ± 2.43 % at *T*_*1*_, for sub-acute; 15.61 ± 3.38 % at *T*_*0*_ and 11.87 ± 2.18 % at *T*_*1*_ for chronic; ***E***_*I*_^*(S)*^: 10.70 ± 1.18 % at *T*_*0*_ and 13.88 ± 1.05 % at *T*_*1*_ for sub-acute; 16.65 ± 1.48 % at *T*_*0*_ and 11.71 ± 2.60 % at *T*_*1*_ for chronic).

**Fig. 3:**
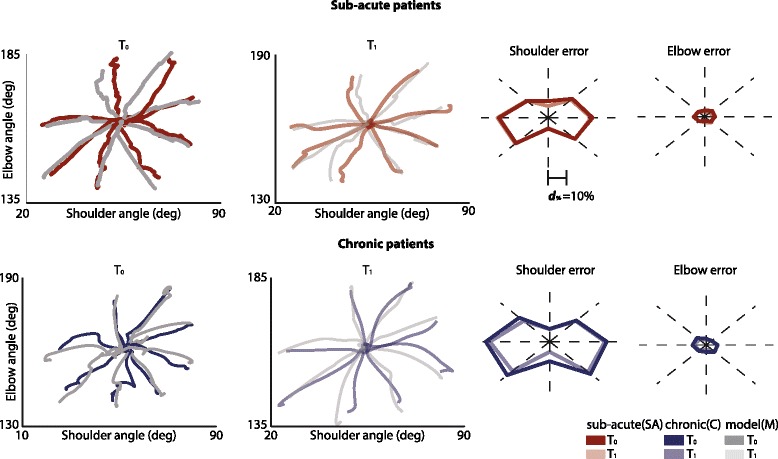
Real and simulated trajectories: Joint space. In the first row the results for the joint angular excursions for a sub-acute representative patient. In particular, in the first and second columns inter-joint coordination between elbow and shoulder angles for the 8 directions of movements, only for movements from the center of the workspace to targets, for *T*_*0*_ (*first column*) and *T*_*1*_ (*second column*) for real (*red lines*) and simulated trajectories (*grey lines*). Angular plots show shoulder (*third column*) and elbow (*fourth column*) normalized distance between real and simulated trajectories for the 8 directions of movements for *T*_*0*_ (*dark colors*) and *T*_*1*_ (*light colors*). In the second row results for a representative chronic patient, same organization of sub-acute patient

Finally the similarity between real and simulated angular excursions (elbow and shoulder) was assessed (see Fig. 3). The low ***d***_***%***_ values between simulated and real angular trajectories (19 % for the shoulder and 5 % for the elbow, on average) showed that joint movements of post-stroke subjects were also finely reproduced.

Taken together these results demonstrated that the proposed computational model was able to simulate trajectories similar to those observed in post-stroke subjects at different impairment levels, both in the Cartesian and in the joint spaces.

### Model-based parameters represents kinematic features of post-stroke trajectories

**Fig. 4:**
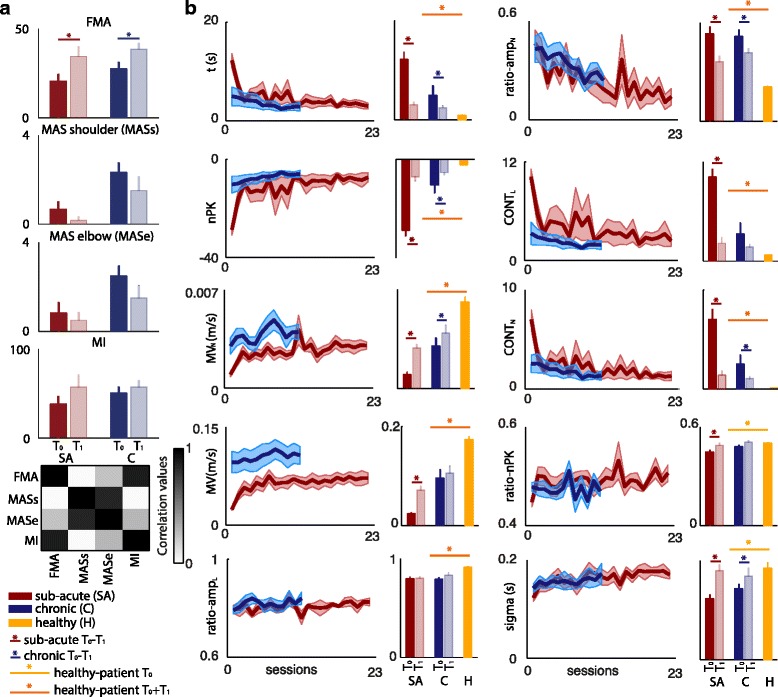
Clinical scores and movement characterization. **a** Mean and standard deviation of clinical scores (FMA, MAS shoulder and MAS elbow, and MI) for *T*_*0*_ and *T*_*1*_ (*dark and light colors bars, respectively*) for sub-acute and chronic patients (*red and blue bars, respectively*). Correlation matrix among clinical scales is also reported. **b** For the *Model-based parameters*, the average ± standard error (*shaded area*) time course of recovery for sub-acute (*red line*) and chronic patients (*blue line*) is reported. In the bar plots mean values (*over repetitions, movement directions, and subjects*) for *T*_*0*_ (*dark colors*) and *T*_*1*_ (*light colors*) for sub-acute (*red bars*) and chronic (*blue bars*) patients and for healthy subjects (*yellow bars*). Standard error is calculated over subjects. Asterisks (*) indicate significant differences (Wilcoxon signed-rank test, *p* < 0.05) between *T*_*0*_ and *T*_*1*_ for sub-acute (*red*), chronic (*blue*). Yellow and orange asterisks (*) refer to significant differences (Mann–Whitney U-test, *p* < 0.05) between post-stroke and healthy subjects for only *T*_*0*_ and for *T*_*0*_ and *T*_*1*_, respectively

Although measuring motor improvements by means of clinical scales was not one of the main aims of this study we reported the scores of both sub-acute and chronic patients after therapy for the purpose of observing parallel significant improvements in both clinical scales and kinematic measures from point-to-point upper limb movements (Fig. 4a). The clinical scores partially correlated. In particular MAS score for the shoulder and the elbow correlated significantly (*r* = 0.85, *p* < 0.001), as well as FMA score and Motricity index (*r* = 0.88, *p* < 0.001). Moderate correlation between the ability to perform isolated joint movements (FMA score) and upper-limb strength (Motricity index) and, in contrast, low correlation with MAS score (*r* = 0.14, *p* >0.05), were in agreement with previous work [6, 32].

We quantified movements’ improvements by computing both the *Model-based* and the *Evaluation parameters* on patients’ trajectories (Fig. 4b and Additional file 3: Figure S3).

Subjects’ movement duration **t**, significantly diminished both for sub-acute and chronic patients across training sessions (*p* < 0.05). The movement smoothness (**nPK**) and **MV**_**N**_, instead, increased significantly along the treatment in both groups, whereas **MV** only in sub-acute patients (*p* < 0.05). Consistently with the increase of the mean velocity, a slight increment in longitudinal peaks amplitude (**ratio-amp**_**L**_) and a significant decrease in transversal peaks amplitude (**ratio-amp**_**N**_) were observed (*p* < 0.05), indicating a progressive translation of submovements toward the longitudinal direction. The increase in movement smoothness, instead, was reflected in a significant reduction of both longitudinal (**CONT**_**L**_, *p* < 0.05 for sub-acute patients) and transversal peaks contribution (**CONT**_**N**_, *p* < 0.05 both for sub-acute and chronic patients). Interestingly, **ratio**-**nPK** had only a slight increase during the therapy, but it reached values comparable to those observed in the healthy subjects. Finally, the significant rise in both patient groups of the **σ** values (*p* < 0.05) revealed an increment of submovements’ duration, which, together with improved smoothness and increased **overlap** (Additional file 3: Figure S3), was consistent with the proposed mechanism of submovements blending during recovery [3]. All parameters values except **ratio**-**nPK** and **<σ>** were significantly different (*p* < 0.003) between post-stroke and healthy subjects at *T*_*0*_ and *T*_*1*_, showing that albeit there were significant motor improvements in upper limb movements, the patients’ recovery was not complete.

We then investigated whether the kinematic features of real trajectories (and their evolution during the rehabilitation process) were well reproduced by simulated trajectories by comparing the values of the *Evaluation parameters* calculated on both trajectory types at *T*_*0*_ and *T*_*1*_ (*Model-based parameters* were used for the generation of the simulated trajectories as explained in section 2.3, and could not be used for the purpose of model evaluation). The *Evaluation parameters* were chosen because they were not directly linked to the *Model-based parameters* by means of known mathematical relationships. Indeed, only **nPK**_**x**_**–pos** and **nPK**_**x**_**–neg** (with *x* ∈ {*L*, *N*}) correlated with **nPK** and **CONT**_**x**_ (on average: *r* = 0.91, *p* < 0.001, Additional file 4: Figure S4A), and **MV**_**L**_, **A, A**_**L**_**–pos**, **A**_**L**_**–neg** with **MV** (on average: *r* = 0.94, *p* < 0.001).

The model was able to reproduce both values and trends (between *T*_*0*_ and *T*_*1*_) in the vast majority of the *Evaluation parameters* (Additional file 3: Figure S3 comparison between gray and colored bars). In particular, movement speed increased, as already showed by the *Model-based parameters* and further supported by the significant increase in **MV**_**L**_, **A**, **A**_**L**_**–pos**, and **A**_**L**_**–neg** for real and simulated trajectories both for sub-acute and chronic patients (*p* < 0.05). Amplitude of transversal peaks (**A**_**N**_**–pos** and **A**_**N**_**–neg**), instead, significantly increased for sub-acute patients and decreased for chronic patients (*p* < 0.05). The simulations also reproduced a raise in the movement smoothness, which was accompanied by a significant reduction of positive and negative peaks both for transversal and longitudinal velocities (**nPK**_**L**_**–pos**, **nPK**_**L**_**–neg**, **nPK**_**N**_**–pos,** and **nPK**_**N**_**–neg**) (*p* < 0.05). Finally, **MD** significantly decreased between *T*_*0*_ and *T*_*1*_ for both patient groups (*p* < 0.05). This decrease could be explained by a translation of the movements toward the longitudinal direction as supported from the increment of **CONT**_**L**_**-amp** and the reduction of **CONT**_**N**_**-amp**.

### Different aspects of motor recovery highlighted by the model-based parameters

**Fig. 5:**
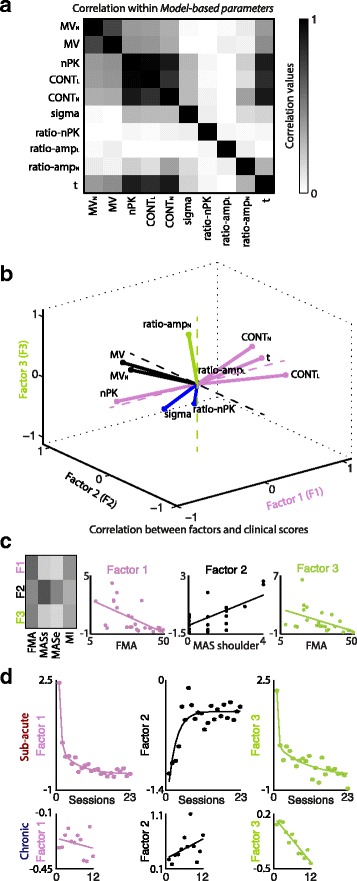
Factorial analysis of *Model-based parameters*. **a** Correlation matrix for the *Model-based parameters*. **b** Three-dimensional factor representation of the *Model-based parameters.* *Pink*, *black*, and *green* lines code the parameters associated with factor 1, factor 2, and factor 3, respectively. *Blue lines* code the parameters that are “shared” across factors. **c** Right: correlation matrix between factors and clinical scores. The colorbar is the same of panel (**a**). MASs and MASe abbreviate MAS shoulder and MAS elbow, respectively. Left: Scatter plots of the correlation between the three main factors and the clinical scores. **d** Temporal evolution of the three factors for sub-acute (*top row*) and chronic patients (*bottom row*). Each point represents the value of the factor for each session averaged across patients. Thick lines represent the results of the fitting of experimental data (i.e., linear, exponential or double exponential). Y-axis scales are different for each factor and patients group

When looking at internal correlations within the *Model-based parameters* (Fig. 5a) we observed that **nPK** was highly correlated with **t** (*r* = 0.94, *p* < 0.001), in agreement with [10], suggesting that submovement blending was strictly intermingled with movement duration shortening. Other parameters, computed on transversal and longitudinal velocities, such as **<σ>**, **ratio-nPK**, **ratio-amp**_**L**_, and **ratio-amp**_**N**_, instead, were not correlated with the others (on average: *r* = 0.24, *p* > 0.05), suggesting that they were likely representing different aspects of motor improvements.

When FA was used to inspect for redundancy among the *Model-based parameters* and to investigate more in detail the different factors involved in motor recovery and particularly which variables measured similar aspects of motor improvement, three main factors were found, explaining together 70 % (52 %, 12 %, and 6 %, respectively) of the total variance (Fig. 5b). The first major factor decreased during the rehabilitation, was related to variables mainly accounting for *movement inefficiency* (**nPK, CONT**_**L**_, **CONT**_**N**_, and **t**)*,* and correlated with FMA score (*r* = 0.60, *p* = 0.002, Fig. 5c). The second factor increased during the rehabilitation, was related to **MV** and **MV**_**N**_ (*movement speed*), and correlated with MAS score for the shoulder (*r* = 0.68, *p* < 0.001). Finally, the third factor correlated with FMA score (*r* = 0.50, *p* = 0.015) and was described by **ratio-amp**_**N,**_ which relates to *movement inaccuracy* as detailed by the high correlation with **MD** (*r* = 0.68, *p* < 0.001).

Interestingly the temporal dynamics during the therapy was different for the three factors (Fig. 5d) suggesting different central and/or peripheral mechanisms concurrently involved in patients’ motor recovery, each with a specific temporal scale. In particular, for sub-acute patients the first and the third factors showed a double-exponential decay, with a comparable fast component (τ ≈ 0.5 and 0.3, respectively), which was even faster than the increase of *movement speed* (τ ≈ 2.5 sessions). The slow component, instead, had a larger decay time constant for *movement inaccuracy* (τ ≈ 10 sessions) than for *inefficiency* (τ ≈ 5.6 sessions), i.e., *inaccuracy* was the slowest to reach a plateau in its temporal evolution during rehabilitation. The best fit for the time courses of the three factors for chronic patients were straight lines. The slopes values revealed a mild decrease for *movement inefficiency* (m ≈ −0.005) over rehabilitation, and a more evident increase for *movement speed* (m ≈ 0.03) and decrease for *inaccuracy* (m ≈ −0.05).

## Discussion

In this study we introduced a computational model of trajectory generation to identify a set of ten variables, the *Model-based parameters*, which allow for an exhaustive kinematic characterization of upper-limb planar movements performed by post-stroke hemiparetic subjects during robot-based training. We then demonstrated that these parameters are informative of three main underlying (and unobserved) recovery factors (*movement inefficiency, speed, and inaccuracy*), each one characterized by a specific time evolution, and, thus, putatively revealing a different central and/or peripheral mechanism of motor restoration.

### A computational model of post-stroke subjects’ trajectories

Previous computational models of trajectory generation in healthy adult subjects include motor control strategies explained by minimum variance [33] minimum jerk [34], minimum torque change [35], and optimal feedback control [36]. However, motor control strategies are often modified by brain insults [37, 38] reflecting the need for tampered computational models. Therefore, in order to generate post-stroke trajectories, we designed a model based on the well-known mechanism of submovement blending [3]. Indeed, in our model, tangential speed profiles of point-to-point movements were assumed to be a sum of submovement curves with bell-shaped Gaussian profiles, i.e., the tangential speed profile was composed of a finite sum of submovements with similar shapes but dilated in duration, translated in time, and modulated in amplitude. A previous work has demonstrated that submovement speed profiles from stroke patients can be appropriately approximated by nearly symmetric β-functions, and that the differences with Gaussian curves were rather minimal [24]. We, therefore, used Gaussian curves for tangential speed and extended this assumption also to transversal and longitudinal velocity profiles. These results would require further experimental validation on a larger population of stroke subjects.

The simulated trajectories generated by our computational model were significantly similar to the real trajectories from post-stroke patients and were able to reproduce the trends in the *Evaluation parameters*. These results demonstrated the capability of the *Model-based parameters* to well characterize the kinematics of patients’ movements and to discriminate between different impairment levels. Additionally, small differences were found between real and simulated angular excursions of limb joints, indicating that impairments at the joint level are influenced by the number and amplitude of peaks in both the transversal and longitudinal speed components. However, the angular excursions at the elbow were better reproduced than those at the shoulder (5 % versus 19 %), probably because of the two-link model limitations to reconstruct shoulder movements normally characterized by three degrees of freedom [25].

Our computational model is currently limited to simulate trajectories of movements autonomously performed by post-stroke subjects which could restrict the applicability of the proposed framework to patients with moderate to mild impairments. Indeed, patients with more severe functional damages most often require robot assistance to accomplish the task [39]. Future works are recommended to address this issue, and to extend the model to other volitional arm movements and assistive devices, such as exoskeletons [40–43]. Indeed, exoskeletons offer several advantages over planar manipulandum because they enlarge the task space to three dimensions following the arm in its natural workspace with no restrictions [44]. Therefore, an extension of our model to three-dimensional trajectories would be of interest for further applications.

Interestingly this model could represent an element of a larger framework for testing new solutions for the reinforcement and the personalization of the therapy [45]. Simulated trajectories could be used to assess whether the patient’s movements follow the expected improvement, thus prompting ‘real-time’ clinical actions. Indeed, the values of *Model-based parameters* computed from real trajectories at *T*_*0*_ and the estimates of motor improvement trends along the rehabilitation training could be fed to the model and used to generate reference trajectories for a given rehabilitation session. Upon comparing these trajectories with the real ones from the patient, actions for task assistance could be immediately triggered, and training refinement could be rightly planned and implemented.

### Model-based kinematic parameters of point-to-point trajectories

Four variables used by the model: **MV nPK**, **<σ>**, and **t**, are well-known parameters proposed by a number of previous studies to characterize motor improvements in post-stroke subjects [5, 39, 46, 47]. They are extracted from the tangential speed profile and they neglect aspects of motor improvement related to end-point accuracy and directional errors. For this reason, they are often complemented by other measures to achieve a more exhaustive description of motor improvement. These additional parameters (*e.g.,* the “mean distance from theoretical path”, the “trajectory straightness”, etc. [47, 48]), however, require *a priori* assumption that point-to-point movements from fully recovered patients, as well as from healthy subjects, are straight, which might be ultimately wrong [49]. Instead, here, we proposed new variables computed from the transversal and longitudinal speed profiles that do not require *a priori* assumptions on movement straightness.

Among these new parameters **MV**_**N**_, **CONT**_**N,**_ and **ratio-amp**_**N**_ were initially considered putative parameters to assess movement accuracy, because they were computed from submovements transversal to the *theoretical path*. However, only **ratio-amp**_**N**_ was highly correlated with **MD**, a well-known measure of movement accuracy [39, 47], whereas **CONT**_**N**_ turned out to be negatively correlated with **nPK**, and **MV**_**N**_ positively correlated with **MV**. The latter was rather unexpected and counterintuitive, because a reduction of average movement speed in the transversal direction was expected, based on the well-documented tendency of post-stroke trajectories to become straighter with training [49]. Instead, **MV**_**N**_ increased in parallel with **MV**, presumably reflecting improved general muscle tone as also demonstrated by the correlation with the MAS score for the shoulder (*r* = 0.50, *p* = 0.002, see Additional file 4: Figure S4B) [11]. The increase of accuracy, instead, was probably obtained by reducing the speed of transversal submovements with respect to longitudinal movements, a complex strategy most likely planned and realized by more central mechanisms involved in motor recovery.

Taken together these new insights significantly contribute to expand the current theory of post-stroke motor recovery based on discrete submovements blending [3].

### Kinematic markers of motor recovery

In our cohort of patients the evaluation of the clinical scales at time *T*_*0*_ and *T*_*1*_ showed that robot-aided therapy led to a reduction of impairment in the hemiparetic limb. This was paralleled by improvements in point-to-point upper limb movements which became progressively similar to those of healthy subjects. We here have to acknowledge the limited sample size of the two patient groups that reduces the strength of the statistical findings. However, the study in which the patients were enrolled was designed as a pilot study [16] with the straight restriction to recruit patients with absence of bilateral impairments.

The application of Factorial Analysis to the *Model-based parameters* showed that the latter were redundant to some extent and informative of three main recovery factors.

The first factor named *movement inefficiency*, was mainly related to variables describing temporal efficiency (movement duration) and movement smoothness. This result confirms and reinforces the quantitative analysis made by Alt Murphy et al. [10] and it advises a reinterpretation of previous disentanglement between these two variables into two different aspects of movement impairments: paresis (*i.e.,* the decreased ability to volitionally modulate motor units activation [13]) and abnormal muscle tone [11]. Moreover, this result suggests that submovement blending, which caused improved movement smoothness, was also one of the main causes of movement duration shortening. The second factor, *movement speed*, was intimately related to muscle tone [11], whereas the third factor (i.e., *movement inaccuracy*) was mainly associated to the decrease of position errors along the primary axis of movement and, thus, to the loss of fractionated movement [11]. The decrease of the fast component of the first and third factors was slightly faster than the increase of *movement speed*. The slow component, instead, was slower for both factors, particularly for *movement inaccuracy*, which was the slowest to reach a plateau in its temporal evolution during rehabilitation. Similar temporal evolutions of the three factors during the rehabilitation process were visible also for chronic patients. However, the best fits for chronic patients were straight lines. Different evolution of the recovery factors could be already expected from the time course of the *Model-based parameters*, which had a linear and exponential evolution for chronic and sub-acute patients, respectively (Fig. 4), and could be related to a more rapid and generalized improvement in early post-stroke period, as suggested in [50], and to a different treatment length.

The importance of the *Model-based parameters* as markers of motor recovery was highlighted also from the correlation with the different clinical scores (see Additional file 4: Figure S4B and 5c). In particular the *Model-based parameters* correlated with the well-accepted FMA score and the MAS score*.* Moreover, as expected from the assessment of the clinical scales, the first and the third factors correlated significantly with the FMA score, while the second factor had a significant correlation with the MAS shoulder score. Indeed, the FMA scale evaluates complex active movements that require the activation of several joints paralleling *motor efficiency*. The MAS scale, instead, evaluates the resistance to passive single-joint movements and it could, thus, relate to the *movement speed*, which decreases with spasticity.

Conclusively the characterization of longitudinal and transversal velocities seems to be essential in clinical application for an exhaustive description of the patients’ motor impairments. The metrics proposed here could be used to complement therapists with immediate measures of motor performance [6] and to design more effective rehabilitation protocols targeted to differentially reduce gap performances in the two orthogonal directions. Interestingly, the existence of correlations among the *Model-based parameters*, and particularly among the parameters related to *movement inefficiency*, and the fact that only three main factors of motor improvement were found by FA point to the possibility to further reduce the number of kinematic variables needed for a computational model to simulate stroke-like trajectories. In this regard, a further study in a larger cohort of patients will be necessary to establish mathematical relationships among the *Model-based parameters*, in order to further reduce their redundancy.

## Conclusions

In this study, we defined a set of kinematic parameters for the characterization of upper limb movements performed by post-stroke hemiparetic subjects during robot-based training. Despite the considerable development of robot-assisted therapy in clinical practice and the various kinematic variables suggested so far, there is still no a general consensus on which parameters to use to evaluate the movement performance. The metrics proposed here are based on a model-driven approach, rather than on specific study hypotheses or on sought relationships with clinical scales. We demonstrated that *i)* they capture relevant kinematic information to assess the quality of reaching trajectories; *ii)* they are manageable, i.e., they do not necessarily require data reduction techniques to extract information about movement performance from a large dataset of computed parameters; *iii)* they reveal diverse factors of kinematic improvement over time, which are informative of different central and/or peripheral mechanisms of motor recovery. By monitoring how these factors change over time at the individual level may provide a new tool to help physiotherapist to take decisions regarding treatment planning.

## Additional files

Additional file 1: Figure S1. Angular plots of the average correlation between longitudinal and transversal components among repetitions of the model and among subjects for the 8 directions of movements for sub-acute patient at *T*_*0*_ (first column) and *T*_*1*_ (second column) and for chronic patients at *T*_*0*_ (third column) and *T*_*1*_ (fourth column). Dark and light gray lines code the values for simulated trajectories at *T*_*0*_ and *T*_*1*_, dark and light red lines code the values for real trajectories of sub-acute patients at *T*_*0*_ and *T*_*1*_, and dark and light blue lines code the values for real trajectories of chronic patients at *T*_*0*_ and *T*_*1*_. For the simulated trajectories, the correlation was in average 0.60 ± 0.09 and 0.55 ± 0.07 for sub-acute patients at *T*_*0*_ and *T*_*1*_, and 0.47 ± 0.07 and 0.62 ± 0.09 for chronic patients at *T*_*0*_ and *T*_*1*_ and was thus comparable to the values of the real trajectories (0.48 ± 0.07 for sub-acute at *T*_*0*_, 0.56 ± 0.08 for sub-acute patients at *T*_*1*,_0.50 ± 0.05 for chronic at *T*_*0*_, 0.49 ± 0.05 for chronic patients at *T*_*1*_). Comparable values were confirmed by statistical tests (Wilcoxon rank sum test, α = 0.05). Indeed, only correlation values for simulated trajectories of chronic patients at *T*_*1*_ were higher than those of the real trajectories (*p* = 0.009). In general, these results show that the 2D correlation between the two components (i.e., longitudinal and transversal) and, thus, the 2D shape of the simulated trajectories was preserved also after decomposition of the tangential velocity. (PDF 413 kb) Additional file 2: Figure S2. Results for the sensitivity analysis for sub-acute patient at *T*_*0*_ (first column) and *T*_*1*_ (second column) and for chronic patients at *T*_*0*_ (third column) and *T*_*1*_ (fourth column). In the y-axis the *Model-based parameters* and in the x-axis the ten variations equally distributed across the probability distribution of each parameter. The average ***E***_*RS*_ was 13.74 ± 0.54 % at *T*_*0*_ and 12.95 ± 1.43 % at *T*_*1*_, for sub-acute patients, and 16.06 ± 1.23 % at *T*_*0*_ and 13.13 ± 0.97 % at *T*_*1*_, for chronic patients. The ***E***_*RS*_ values were comparable to those found with the parameters values chosen showing that the model is robust for variation of the parameters. (PDF 89 kb) Additional file 3: Figure S3. Evaluation parameters. The bar plots show average and standard error of the *Evaluation parameters* for real trajectories (left bars) and the simulated trajectories (right bars) both for *T*_*0*_ (dark colors) and *T*_*1*_ (light colors). Red and blue colors code sub-acute and chronic patients. Asterisks (*) indicate significant differences (Wilcoxon signed-rank test, *p* < 0.05) between *T*_*0*_ and *T*_*1*_ for sub-acute (red), chronic (blue), and modeled trajectories (grey). (PDF 273 kb) Additional file 4: Figure S4. A) Correlation matrix for *Model-based parameters* and *Evaluation parameters.* B) Correlation matrix for *Model-based parameters* and clinical scores. (PDF 188 kb)

## Abbreviations

FA: Factor analysis
FMA: Fugl-meyer assessment
ICA: Independent component analysis
MAS: Modified ashworth scale
MI: Motricity index
PCA: Principal component analysis
