# Supplementary material for: Model-based variables for the kinematic assessment of upper-extremity impairments in post-stroke patients
Source: J Neuroeng Rehabil. 2016 Sep 8;13(1):81. doi: 10.1186/s12984-016-0187-9 (PMC5016877; doi:10.1186/s12984-016-0187-9)
Supplement: Additional file 1: Figure S1. — Angular plots of the average correlation between longitudinal and transversal components among repetitions of the model and among subjects for the 8 directions of movements for sub-acute patient at T 0 (first column) and T 1 (second column) and for chronic patients at T 0 (third column) and T 1 (fourth column). Dark and light gray lines code the values for simulated trajectories at T 0 and T 1, dark and light red lines code the values for real trajectories of sub-acute patients at T 0 and T 1, and dark and light blue lines code the values for real trajectories of chronic patients at T 0 and T 1. For the simulated trajectories, the correlation was in average 0.60 ± 0.09 and 0.55 ± 0.07 for sub-acute patients at T 0 and T 1, and 0.47 ± 0.07 and 0.62 ± 0.09 for chronic patients at T 0 and T 1 and was thus comparable to the values of the real trajectories (0.48 ± 0.07 for sub-acute at T 0, 0.56 ± 0.08 for sub-acute patients at T 1,0.50 ± 0.05 for chronic at T 0, 0.49 ± 0.05 for chronic patients at T 1). Comparable values were confirmed by statistical tests (Wilcoxon rank sum test, α = 0.05). Indeed, only correlation values for simulated trajectories of chronic patients at T 1 were higher than those of the real trajectories (p = 0.009). In general, these results show that the 2D correlation between the two components (i.e., longitudinal and transversal) and, thus, the 2D shape of the simulated trajectories was preserved also after decomposition of the tangential velocity. (PDF 413 kb) [file 12984_2016_187_MOESM1_ESM.pdf]

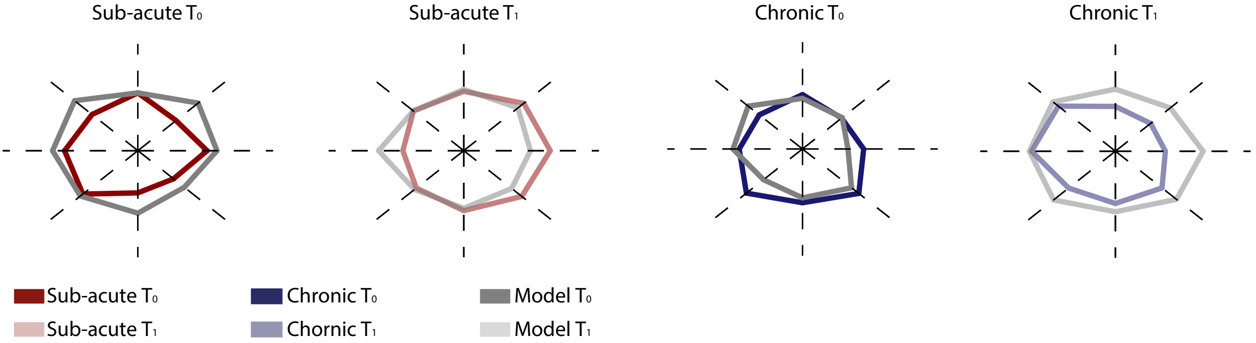

Sub-acute T0
Sub-acute T1
Chronic T0
Chronic T1
Sub-acute T0
Sub-acute T1
Chronic T0
Chornic T1
Model T0
Model T1
