# Supplementary figures and images for: Model-based variables for the kinematic assessment of upper-extremity impairments in post-stroke patients
Source: J Neuroeng Rehabil. 2016 Sep 8;13(1):81. doi: 10.1186/s12984-016-0187-9 (PMC5016877; doi:10.1186/s12984-016-0187-9)

Sub-acute  $T_0$ Sub-acute  $T_1$ Chronic  $T_0$ Chronic  $T_1$ 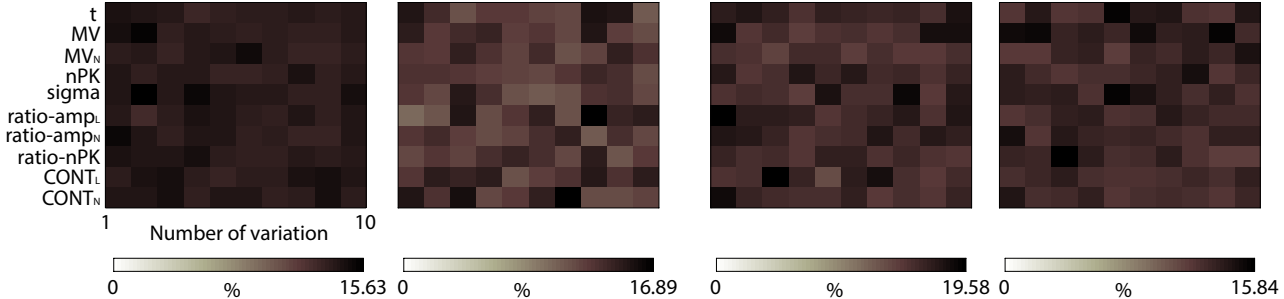

Supplement: Additional file 2: Figure S2. — Results for the sensitivity analysis for sub-acute patient at T 0 (first column) and T 1 (second column) and for chronic patients at T 0 (third column) and T 1 (fourth column). In the y-axis the Model-based parameters and in the x-axis the ten variations equally distributed across the probability distribution of each parameter. The average E RS was 13.74 ± 0.54 % at T 0 and 12.95 ± 1.43 % at T 1, for sub-acute patients, and 16.06 ± 1.23 % at T 0 and 13.13 ± 0.97 % at T 1, for chronic patients. The E RS values were comparable to those found with the parameters values chosen showing that the model is robust for variation of the parameters. (PDF 89 kb) [file 12984_2016_187_MOESM2_ESM.pdf]

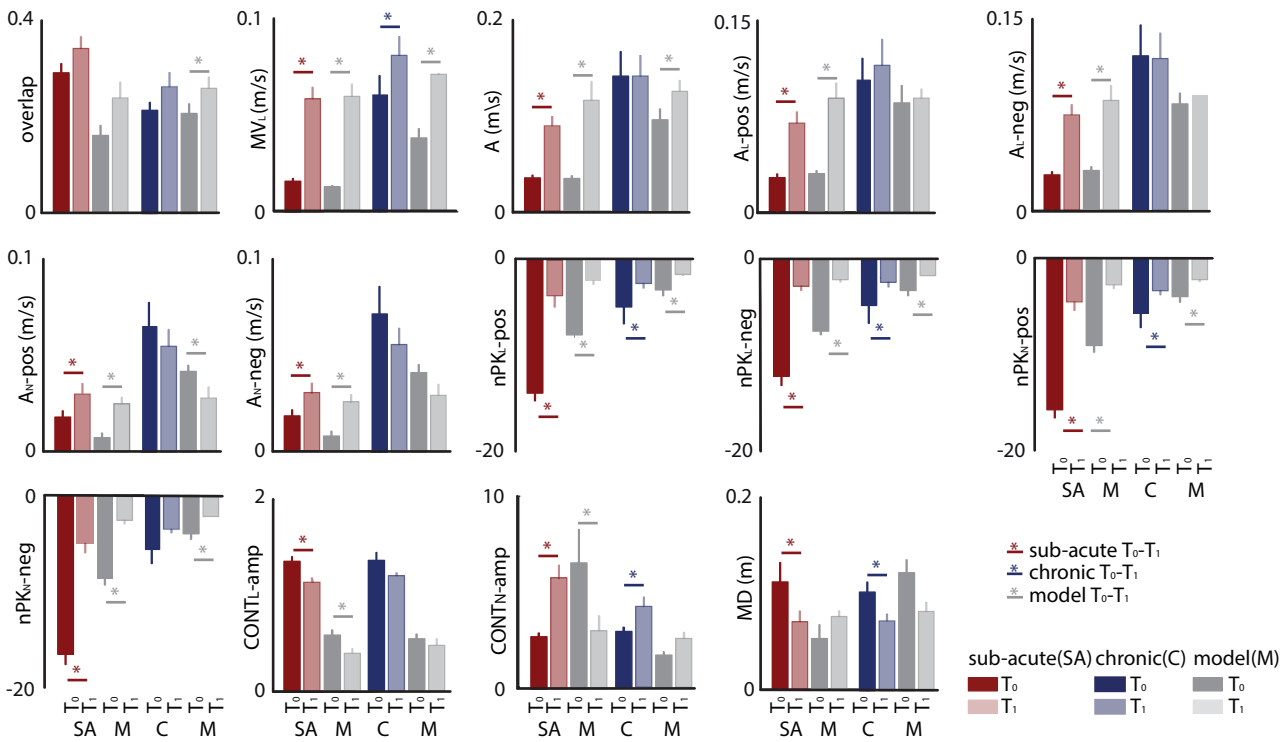

Supplement: Additional file 3: Figure S3. — Evaluation parameters. The bar plots show average and standard error of the Evaluation parameters for real trajectories (left bars) and the simulated trajectories (right bars) both for T 0 (dark colors) and T 1 (light colors). Red and blue colors code sub-acute and chronic patients. Asterisks (*) indicate significant differences (Wilcoxon signed-rank test, p < 0.05) between T 0 and T 1 for sub-acute (red), chronic (blue), and modeled trajectories (grey). (PDF 273 kb) [file 12984_2016_187_MOESM3_ESM.pdf]

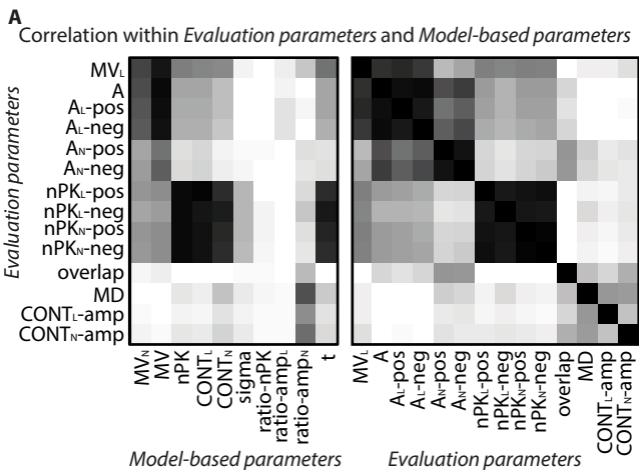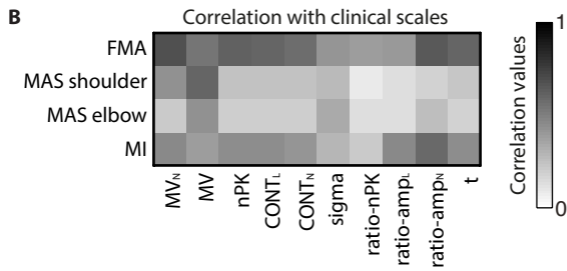

Supplement: Additional file 4: Figure S4. — A) Correlation matrix for Model-based parameters and Evaluation parameters. B) Correlation matrix for Model-based parameters and clinical scores. (PDF 188 kb) [file 12984_2016_187_MOESM4_ESM.pdf]
